# Coffee consumption and risk of endometrial cancer: a dose-response meta-analysis of prospective cohort studies

**DOI:** 10.1038/srep13410

**Published:** 2015-08-25

**Authors:** Quan Zhou, Mei-Ling Luo, Hui Li, Min Li, Jian-Guo Zhou

**Affiliations:** 1 Department of Science and Education, First People’s Hospital of Changde City, Changde 415003, Hunan, People’s Republic of China; 2 Changsha Center for Disease Control and Prevention, Changsha 410001, Hunan, People’s Republic of China; 3 Department of Oncology, First People’s Hospital of Changde City, Changde 415003, Hunan, People’s Republic of China; 4 Department of Neurology, Second Affiliated Hospital of Nanchang University, Nanchang 330006, Jiangxi, People’s Republic of China; 5 Department of Oncology, Affiliated Hospital of Zunyi Medical University, Address: NO.149, Dalian Road, Zunyi City, Guizhou Province, China, 653000

## Abstract

This is a dose-response (DR) meta-analysis to evaluate the association of coffee consumption on endometrial cancer (EC) risk. A total 1,534,039 participants from 13 published articles were added in this meta-analysis. The RR of total coffee consumption and EC were 0.80 (95% *CI*: 0.74–0.86). A stronger association between coffee intake and EC incidence was found in patients who were never treated with hormones, 0.60 (95% *CI*: 0.50–0.72), and subjects with a BMI ≥25 kg/m^2^, 0.57 (95% *CI*: 0.46–0.71). The overall RRs for caffeinated and decaffeinated coffee were 0.66 (95% *CI*: 0.52–0.84) and 0.77 (95% *CI*: 0.63–0.94), respectively. A linear DR relationship was seen in coffee, caffeinated coffee, decaffeinated coffee and caffeine intake. The EC risk decreased by 5% for every 1 cup per day of coffee intake, 7% for every 1 cup per day of caffeinated coffee intake, 4% for every 1 cup per day of decaffeinated intake of coffee, and 4% for every 100 mg of caffeine intake per day. In conclusion, coffee and intake of caffeine might significantly reduce the incidence of EC, and these effects may be modified by BMI and history of hormone therapy.

EC is a common type of gynaecologic cancer. The overall cases reported each year is third in rank among the number of new cases and one of the major (4^th^ Rank) cause of deaths in the female around the world^1^. In the developed countries, the incidence is more than two fold than in developing countries^2^. In United States (US), it is anticipated that approximately 54870 women will be diagnosed with EC in 2015^3^. Coffee is commonly consumed among all the beverages globally. More than 2.5 billion cups of coffee are consumed worldwide each day^4^. It contains various phytochemicals having potential antioxidant and anti-mutagenic properties^5^ and minor health effects may lead to impact the public health in large. Recently, the connection between coffee consumption and associated risk of cancer has gained great interest^6^,^7^,^8^. As per EC report from the World Cancer Research Fund/American Institute for Cancer Research (WCRF/AICR), coffee is a protective factor against EC^9^.

As per our current knowledge, only four published meta-analyses were focused on the involvement of coffee for and EC risk^10^,^11^,^12^,^13^. In a meta-analysis, 2009^13^, it was found an inverse association between high coffee consumption and EC. In another meta-analysis^10^ of four cohort studies, it has been reported RR of 0.74 between high *vs*. low coffee intake group. Recently, an article^11^ that combined a cohort study and meta-analysis was published, and a weak relation between coffee consumption and EC was found. However, these four meta-analyses did not further explore the relationship between different caffeine dosages or coffee types, and graph of DR relationship. Moreover, recent meta-analysis^11^ did not include four large cohort studies, i.e. approximately 2000 cases and 369624 participants were ignored.

Therefore, we designed this meta-analysis with the following objectives: (1) to investigate the epidemiological evidence on the relationship between total coffee intake and EC risk. It also quantifies the association between different caffeine dosages and coffee types and risk of EC; (2) to calculate the DR graph of these associations; and (3) to evaluate the statistical power of the associations and the quality of evidence.

## Methods and Materials

This meta-analysis was designed as per the Meta-analysis of Observational Studies in Epidemiology (MOOSE) guidelines^14^.

### Date source and search strategy

The PubMed, Embase and the Cochrane Library databases were searched from inception to May 1, 2015 for studies describing the association between coffee consumption and incidence of EC. The key words used during searching were : (“endometrial cancer” OR “endometrium cancer” OR “endometrial carcinoma” OR “endometrium carcinoma” OR “endometrial neoplasms” OR “endometrium neoplasms” OR “corpus uteri cancer” OR “corpus uteri neoplasm” OR “corpus uteri carcinoma” OR “uterus tumor” OR “uterine tumor”) AND (“Coffee” OR “coffee” OR “coffee consumption” OR “coffee intake” OR “coffee drinking” OR “Caffeine” OR “caffeine consumption”). In addition, the references from applicable research papers and review papers were screened to identify studies. In case of duplicated data, the most complete study was only considered for this Meta analysis.

### Study selection and data extraction

The selection of titles and abstracts of the studies were done as the first step for scrutinization. The second selection was done based on a complete review of the paper. The studies meeting the following conditions were only included in this study:

(1) Study design was a prospective cohort study;

(2) Exposure of interest was coffee intake;

(3) Outcome of interest was EC;

(4) RR or HR and the corresponding CI of the EC for the maximum versus the minimum level were provided.

The RRs or HRs had to be adjusted for potential confounders. Two independent investigators (QZ and ML-L) selected the studies. If the studies did not not meet the all the above criteria, were excluded from this analysis. From the selected studies the information isolated were: first author’s last name; publication year; country of origin; study period; cohort name; duration of follow-up; age at baseline; coffee intake category; number of cases and cohort size of each category;relative risks (with their 95% CI) for each category of coffee consumption, and covariates adjusted for in the multivariable analysis. Two investigators (QZ and HL) independently retrieved the data. This disagreement in the independent analysis was resolved by mutual agreement.

### Quality assessment of studies and evidence

The Newcastle-Ottawa Scale^15^ (NOS) were used to assess the quality of the included studies by two reviewers (QZ and ML). This scale ranged from 0 (poor) to 9 stars (excellent) and awards four stars for selection of study participants, two stars for comparability of studies, and three stars for the adequate ascertainment of outcomes. Scores of 0–3, 4–6, and 7–9 are regarded as low, moderate, and high quality, respectively^16^. The quality of evidence was evaluated using the GRADE system^17^,^18^ (GRADE profiler 3.6.1) by two reviewers (QZ and JG-Z). There are several reasons for downgrade of data which are; inconsistency, indirectness, imprecision or publication bias, and three reasons to upgrade the evidence: large effect size, dose-response relationship and plausible confounders that would not decrease the apparent effect size^19^. The categories for the different evidences are: High; Moderate; Low and very low.

### Statistical analysis

Because incidence of EC is low, HR is mathematically similar to RR^20^. The results were reported as RR. We quantified the associations between total coffee caffeine dosage, caffeinated coffee, decaffeinated coffee intake and risk of EC comparing the highest versus the lowest (the referent) categories. For the dose-response meta-analysis, The dosage value assigned to each coffee intake category is the median provided by the original research.For the studies that did not report a median, we used the midpoint for closed categories. In case of open ended highest category, the midpoint of the category was set at 1.5 times the lower boundary and in case of open ended lowest category, the lower boundary was set to zero^21^. The least squares estimation was done by Greenland and Longneck method^22^,^23^ to account for the correlation with the log RR estimates across the exposures. The potential DR relationship was estimated in two stages^24^; firstly, restricted cubic spline model with 4 knots at percentiles 5%, 35%, 65% and 75% of the distribution of exposure consumption was estimated, and the 3 regression coefficients (4 knots minus 1) were calculated; Secondly, the variance or covariance matrix within each study was combined. Non-linearity test was made by testing the null hypothesis that both the coefficients of the second and third spline are all equal to zero.If the p value more than 0.05 was considered as linear and less than 0.05 was considered as nonlinear. Heterogeneity among studies was assessed using the *χ*^*2*^ test and was defined as a *P* value less than 0.10. Quantification of heterogeneity was assessed by the *I*^*2*^statistic^25^. An *I*^*2*^ above 50% indicated high heterogeneity. A random effect was implemented^26^. Publication bias was judged with the Begg rank correlation test^27^ and Egger’s regression test^28^. The results were considered to show publication bias when p < 0.10. If publication bias existed, we used a trim and fill method^29^ to evaluate the number of missing studies. A stratified analysis was performed for study, location, BMI, smoking status, hormone therapy, menopausal status, and interval of follow-up, total of participants, number of cases and results were adjusted whether for BMI or hormone therapy. A power calculation was performed using the method described by Cafri *et al.*^30^. Highest versus lowest meta-analysis and subgroup analysis were performed in Review Manager Version 5.3 (The Nordic Cochrane Center, Copenhagen, Denmark). The sensitivity analyses were estimated in Stata version 12.0 (StataCorp, College Station, Texas, USA). The trim and fill method was conducted in R version 3.2.0 (R Foundation for Statistical Computing, Vienna, Austria). A signifance level is said when the *p* was less than 0.05, unless otherwise specified.

## Results

### Literature search

**Figure 1:**
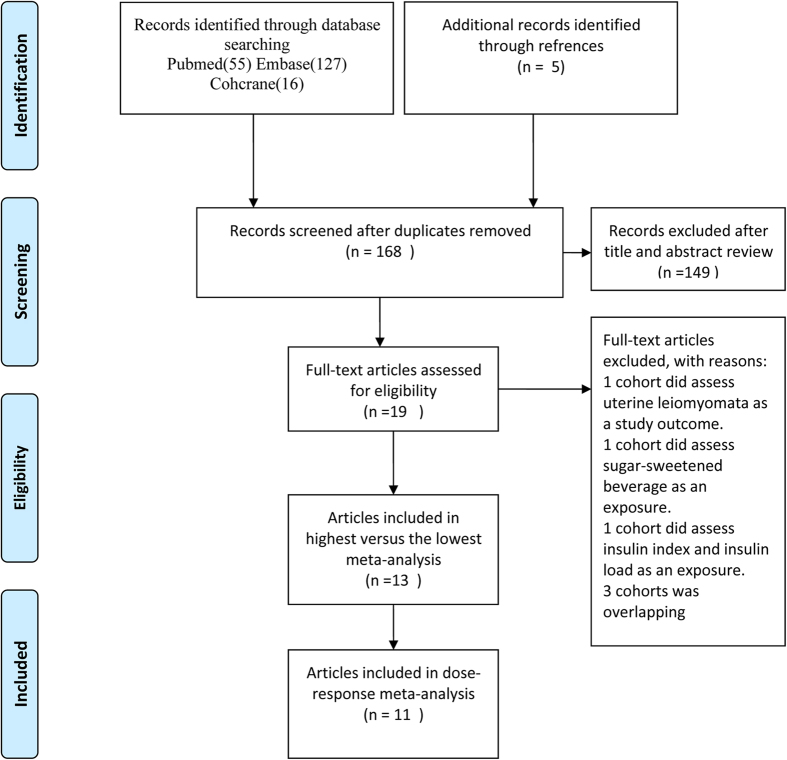
Flowchart of literature search and study selection.

The Fig. 1 shows the flow chart for literature retrieval and selection. In first search a total 198 published articles from PubMed, Embase and Cochrane Library prior to May 1, 2015 were identified. 5 associated papers were also identified by the manual literature search. All the identified articles were screened and, after screening only 19 papers were selected. From these 19 papers, 6 studies were excluded due to following reasons:

(1) One study^31^ assessed uterine leiomyomata as a study outcome;

(2) Two studies^32^,^33^, did not assess coffee intake;

(3) Three^34^,^35^,^36^ were overlapping cohorts.

Finally 13 articles^11^,^37^,^38^,^39^,^40^,^41^,^42^,^43^,^44^,^45^,^46^,^47^,^48^ were selected and these papers were included this meta-analysis. Among these articles, one study provided information both on type I and type II EC. Thus; our main meta-analysis were done in 14 comparisons obtained from 13 studies.

In the DR meta-analysis, we excluded papers of Jacobsen *et al.*^43^ and Merritt *et al.*^48^ because in the first paper only two levels of coffee intake categorised and in the second paper coffee intakes were calculated at each center in grams/day to account for differences in cup sizes by region. Finally, the DR meta-analysis was done in 12 comparisons from 11 studies only^11^,^37^,^38^,^39^,^40^,^41^,^42^,^44^,^45^,^46^,^47^.

### Study characteristics

The Table 1 shows the characteristics of all the selected studies. All 13 selected studies were prospective cohort with subjects without EC at baseline. A total 1,534,039 participants were included in this study. A total of 10,100 cases of EC were documented during the follow-up period of 11–20 years. From the total studies eight studies^11^,^37^,^39^,^41^,^43^,^46^,^47^,^48^ were conducted in Europe, three^38^,^42^,^44^,^45^ in the USA and one in Asia (Japan^40^). The maximum numbers (560,356) of participants were from UK Million Women Study^11^ and minimum numbers (2891) were from Norwegian cohorts^43^. The participant’s age at baseline ranged from 30 to 79 years. Only three studies^38^,^45^,^46^ included postmenopausal women, and other studies^11^,^37^,^39^,^40^,^41^,^42^,^43^,^44^,^47^,^48^ included both premenopausal and postmenopausal women. For the assessment of dietary coffee intake, the Nurses’ Health Study (NHS^42^) used seven food frequency questionnaires (FFQs), whereas the remaining studies used one^11^,^37^,^38^,^39^,^43^,^46^,^48^ or two^41^,^45^,^47^ FFQs. Four papers^38^,^42^,^44^,^45^ (five comparisons) provided information on caffeinated coffee intake, and three articles^37^,^38^,^42^ (four comparisons) measured the total caffeine intake per day and the risk of EC. All studies provided adjusted risk estimates, the RRs were adjusted for age (12 studies), BMI (11 studies), smoking (12 studies) and use of exogenous hormones (9 studies). NOS scores ranged from six to nine. The most common reason for deductions was an insufficient adjustment for the potential confounders^39^,^41^,^43^,^46^,^47^ (Supplementary Appendix 1).

### Highest versus lowest meta-analysis

**Figure 2:**
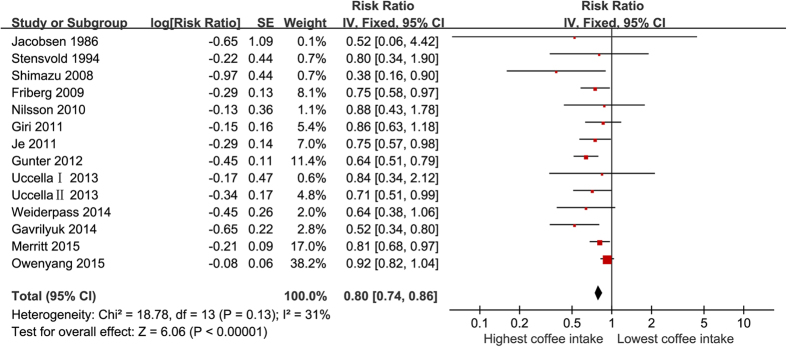
Forest plot of total coffee intake and relative risk of EC.

**Figure 3:**
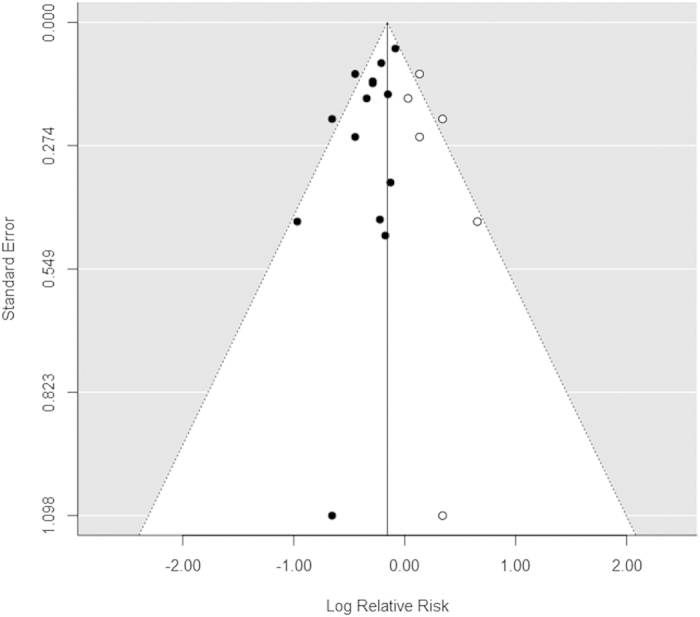
Filled funnel plot of total coffee intake and relative risk of EC.

Fourteen comparisons from thirteen^11^,^37^,^38^,^39^,^40^,^41^,^42^,^43^,^44^,^45^,^46^,^47^,^48^ studies had a link between coffee ingestion and EC risk. The summary of RR for the highest and lowest categories was 0.80 (95% *CI*: 0.74–0.86) (Fig. 2). Heterogeneity among studies was not statistically significant (*P *= 0.13, *I*^2^ = 31%). Egger regression tests (*P *= 0.03) were used to showed publication bias, but not by Begg correlation test (*P *= 0.66). The estimate was found to be 0.86 (0.80–0.91) after adjusting the six missing studies (Fig. 3). Four comparisons from three studies^37^,^40^,^46^ provided the caffeine dose per day and EC risk. The summary RR of the highest versus the lowest categories was 0.77 (95% *CI*: 0.65–0.92).Heterogeneity among studies was not statistically significant (*P *= 0.87, *I*^2^ = 0%) and both Egger regression test(*P *= 0.61) and Begg correlation test (*P *= 0.75) did not detected publication bias. Five comparisons of the four studies^38^,^42^,^44^,^45^ reported a link between different types of coffee and EC risk. The summary RR for caffeinated coffee was 0.66 (95% CI: 0.52–0.84) along with evidence of heterogeneity (*P *= 0.07, *I*^2^ = 53%). As such Begg correlation test (*P *= 0.23) or the Egger regression test (*P *= 0.47) showed no evidence of substantial publication bias. For the decaffeinated coffee, the summary RR was 0.77 (95% *CI*: 0.63–0.94). No heterogeneity (*P *= 0.76, *I*^2^ = 0%) and publication bias among studies indicated by the Begg correlation test (*P *= 1.00) and the Egger regression test (*P *= 0.88). Tests for subgroup differences showed no significant differences between caffeinated and decaffeinated coffee (*P *= 0.35) (Table 2).

### Subgroup and sensitivity analysis

The subgroup analyses are shown in the Table 3. The observed inverse association was more pronounced in the group of BMI more than 25 kg/m^2^ (RR = 0.57, 95% *CI*: 0.46–0.71) and in those without hormone therapy (RR = 0.60, 95% *CI*: 0.50–0.72). However, no association was observed in the group of BMI less than 25 kg/m^2^ (RR = 0.99, 95% *CI*: 0.86–1.15) or among hormone therapy participants (RR = 0.85, 95% *CI*: 0.65–1.11). Sensitivity analyses results are in the range from 0.73 (95% *CI*: 0.67–0.81) to 0.82 (95% *CI*: 0.76–0.89) for total coffee intake.

### DR analysis

**Figure 4:**
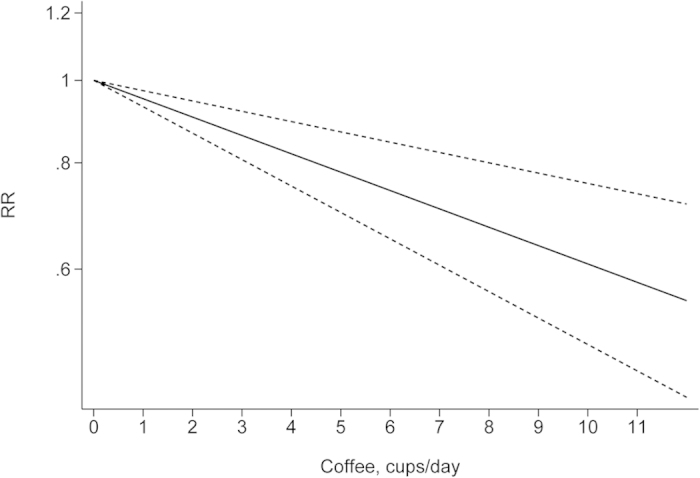
Dose-response analysis of total coffee intake and relative risk of EC.

**Figure 5:**
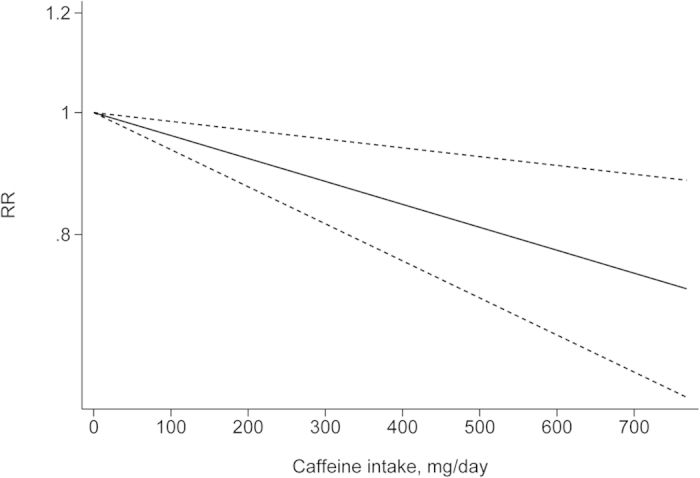
Dose-response analysis of caffeine intake per day and risk of EC.

**Figure 6:**
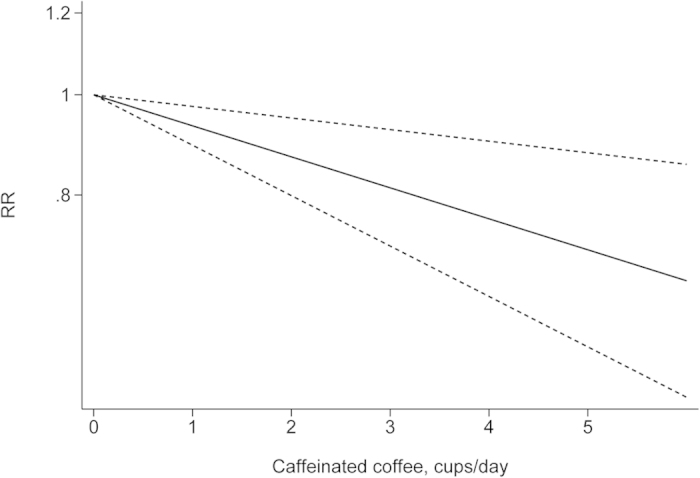
Dose-response analysis of caffeinated coffee intake and risk of EC.

**Figure 7:**
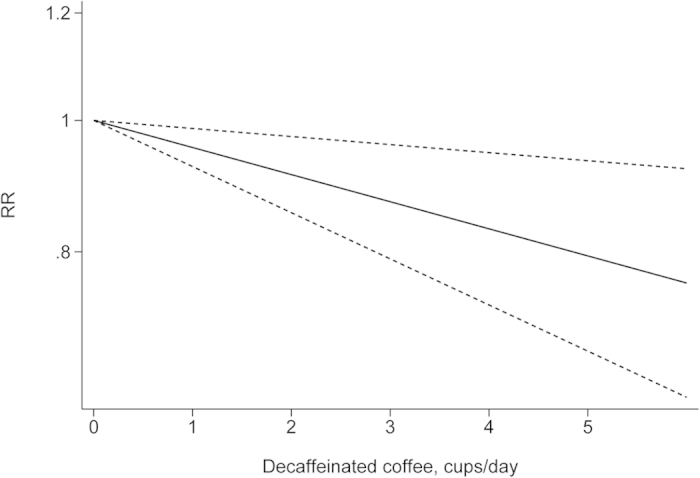
Dose-response analysis of decaffeinated coffee intake and risk of EC.

The departure from nonlinearity was not significant (*P *= 0.90), and an increase of 1 cup of coffee per day was associated with a 5% (RR = 0.95, 95% *CI*: 0.93–0.97) lower risk of EC (Fig. 4). Four comparisons from three studies^37^,^38^,^42^ included DR analysis of caffeine intake per day and risk of EC. The test for nonlinearity (*P *= 0.89) supported a linear model and indicated that an increased intake of 100 mg caffeine per day was associated with a 4% (RR = 0.96, 95% *CI*: 0.93–0.98) lower risk of EC (Fig. 5). Five comparisons from four studies^42^,^44^,^45^,^46^ included the DR analysis of different types of coffee and risk of EC. For caffeinated coffee, the test for nonlinearity was not significant (*P *= 0.82), and the results showed that increased intake of 1 cup of caffeinated coffee per day was associated with a 7% (RR = 0.93, 95% *CI*: 0.89–0.97) lower risk of EC (Fig. 6). For decaffeinated coffee, the test for nonlinearity was not significant (*P *= 0.50), and the results showed that increased intake of 1 cup of decaffeinated coffee per day was associated with a 4% (RR = 0.96, 95% *CI*: 0.92–0.99) lower risk of EC (Fig. 7).

### Power analysis and quality of evidence

Power calculations were performed post hoc as per the method described by Cafri *et al.*^30^. The power to detect an RR of 0.80 for the total coffee intake was 100%, 84.98% for an RR of 0.77 for caffeine intake per day, 92.09% for an RR of 0.66 for caffeinated coffee intake and 72.47% for an RR of 0.77 for decaffeinated coffee intake (Supplementary Appendix 2). RR of total coffee intake and EC was the critical outcome. The others were important outcomes. The evidence of quality for caffeine dosage, decaffeinated coffee intake and risk of EC were moderate. The evidence of quality for total coffee intake, caffeinated coffee intake and risk of EC were low (Table 4).

## Discussion

The possible relationship between the intake of coffee and EC risk was first explored in a Norwegian prospective study in 1986^43^; many cohort studies have attempted to confirm this hypothesis. However, the role of coffee intake in EC is controversial. Six studies^40^,^42^,^44^,^46^,^47^,^48^ have found a decreased risk of EC with high coffee intake, but five studies^11^,^37^,^39^,^41^,^45^ failed to find a significant association. Additionally, in an Iowa Women’s Health Study (IWHS)^38^, a significant inverse association was found only with Type I EC. The intake of total coffee, caffeine, and caffeinated and decaffeinated coffee was inversely associated with EC in a highest versus lowest meta-analyses. The intake of caffeine results in the percentage decrease in risk of EC, which was shown by DR analyses. This study indicated a 5% lower risk of EC by increase the intake of total coffee having each 1 cup/day, a 4% lower risk of EC with each 100 mg/day by an increase in the intake of total caffeine, a 7% lower risk of EC with 1 cup/day by an increase in the intake of caffeinated coffee and a 4% lower risk of EC with each 1 cup/day as we increases the intake of decaffeinated coffee. Our results are consistent with the EC 2013 report of the continuous update project WCRF/AICR^9^. A DR meta-analysis showed a 7% and 8% decreased risk of EC with an increased intake of one cup of total coffee and decaffeinated coffee per day, respectively.

The prospective cohort study design was included in almost all the studies and little heterogeneity was found in our meta-analysis. Except one study in Japan^40^, various studies conducted in Western countries where most of the population shared genetic background, lifestyle, dietary pattern^49^; and the EC incidences in most studies were comparable. Mostly the results of our subgroup analyses were quite similar and robust. There were no differences in study location, smoking status, menopausal status, follow-up length, number of participants and number of cases, but the associations was modified by BMI and history of hormone therapy. The inverse association was more among participants having a BMI greater than 25 kg/m^2^ (RR = 0.57, 95% *CI*: 0.46–0.71) and among participants never received hormone therapy (RR = 0.66, 95% *CI*: 0.550.79). However, whether the study adjusted for BMI or hormone therapy did not affect the results in our study. The statistically non-significant subgroup differences occurred because of the small number of studies, which did not adjust for BMI (n = 2) or hormone therapy (n = 4) such that there was insufficient statistical power.

Several mechanisms were explained for the association of the coffee with EC. Caffeine and methylxanthine in coffee may increase amount of sex-hormone-binding globulin, thus reducing the concentrations of sex-steroids and leading to down regulation of endometrial hyper proliferation^50^. These compounds include phenol compounds, chlorogenic acid, which produces catechins, caffeic, ferulic and coumaric acids that are partly lost during roasting^51^, melanoidins that are mainly produced during roasting and diterpenes that may have anticarcinogenic effects^8^,^52^. In addition, it is also reported that coffee may be an insulin sensitizer^53^. Coffee and caffeine intake were inversely related with intensities of circulating C-peptide, this association was much higher in overweight and obese women^54^.

The current study has several advantages, compared to the previous published meta-analyses^10^,^11^,^12^,^13^. A DR analysis was also conducted to see the possible associations so the results are more reliable. In addition, the association between different caffeine dosages and types of coffee was examined. Furthermore, the statistical power was calculated to assess the probability of correctly rejecting the null hypothesis. In this meta-analysis, the power for the RR value of coffee intake and risk of EC incidence was 100%.

There are several limitations of this study as listed below:

(1) Most of the studies from America and Europe and only one study conducted in Japan. So more studies need to be included from other part of world much as Asia and South Africa.

(2) Coffee intake data were collected using food frequency questionnaires (FFQs). Findings from large population-based studies in Sweden^48^,^55^,^56^ (EPIC cohort) and the US^44^ (NIH-AARP cohort) have shown that coffee intake tends to be stable over a very long period of time.

(3) Most studies did not distinguish between types of EC. Only one study^38^ reported a statistically non-significant protective effect between coffee intake and type II EC. However, recent studies from the University of Southern California have shown that two EC types share many common aetiologic factors. The aetiology of type II tumours may not be completely oestrogen independent, as previously believed^57^.

(4) The possibility of residual confounding due to other risk factors cannot be excluded, although most investigators had adjusted BMI, hormone therapy, and smoking status.

(5) Publication bias was detected in the total coffee intake and EC risk. However, we added six studies with null results trend in the funnel plot, the overall effect size still showed a protective effect.

(6) The GRADE quality of the evidence was moderate or low, which lowers confidence in any subsequent recommendations.

This study meets several of the Hill criteria for causation^58^,^59^: Temporality; Strength; Consistency; Biological gradient; Plausibility.

In summary, coffee, regardless of coffee type and caffeine dosage, is a potential protective factor against EC in a dose-dependent fashion; the results corroborate the findings of the conclusion of the EC 2013 Report by WCRF/AICR. Further, the association of coffee intake and EC risk may be adapted by BMI and history of hormone therapy.

## Additional Information

**How to cite this article**: Zhou, Q. *et al.* Coffee consumption and risk of endometrial cancer: a dose-response meta-analysis of prospective cohort studies. *Sci. Rep.* **5**, 13410; doi: 10.1038/srep13410 (2015).

## Supplementary Material

Supplementary Information

## Figures and Tables

**Table 1:** Characteristics of prospectivestudies included that assessed the association between coffee intake and EC.

**Reference**	**Country**	**Cohort Name**	**Age at baseline**	**Follow up**	**Cases/Cohort Size**	**NOS**	**Highest vs. lowest (Coffee intake)**	**RR (95% CI)**	**Adjustments**
Jacobsen,1986	Norway	Norwegian cohorts	NA	11.5	11/2891	7	≥7 cups/day vs. ≤2 cups/day	0.52(0.06–4.44)	age, residence
Stensvold,1994	Norway	Norwegian prospective study	35–54	10.1	84/21238	6	≥7 cups/day vs. ≤2 cups/day	0.8(0.34–1.90)	age, cigarettes per day, county of residence
Shimazu,2008	Japan	Japan Public Health Center-based Prospective Study	40–69	15	117/53724	9	≥3 cups/day vs. ≤2 cups/week	0.38(0.16–0.91)	age, study center, BMI, menopausal status, age at menopause, use of exogenous hormones, smoking status, green vegetable consumption, beef consumption, pork consumption, green tea consumption
Friberg,2009	Sweden	Swedish Mammography Cohort	40–76	17.6	677/60634	6	≥4 cups/day VS.≤1 cups/day	0.75(0.58–0.97)	age in months, BMI and smoking
Nilsson,2010	Sweden	Vasterbotten Intervention Project (VIP)	50	15	108/30639	7	≥4 cups/day vs. <1 cups/day	0.88(0.44–1.78)	age, BMI, smoking, education, and recreational physical activity
Giri,2011	USA	Women’s Health Initiative (WHI) Observational Study	50–79	7.5	427/45696	8	≥4 cups/day vs. 0 or <1 cup/day	0.86(0.63–1.18)	age, ethnicity, unopposed oestrogen use, progestin + oestrogen use, smoking and BMI
Je,2011	USA	Nurses’Health Study (NHS)	30–55	26	672/67470	7	≥4 cups/day vs. <1cup/day	0.75(0.57–0.97)	age, BMI, age at menopause, age at menarche, parity and age at last birth, parity, duration of oral contraceptive use, postmenopausal hormone use, pack-years of smoking, alcohol intake and total energy intake
Gunter,2012	USA	The NIH-AARP Diet and Health Study	50–71	9.3	1486/226732	7	>3cups/day vs. 0 cups/day	0.64(0.51–0.80)	age, smoking, BMI, age at menarche, age at first child’s birth, parity, age at menopause, HT use, oral contraceptive use, diabetes and physical activity
Uccella1,2013	USA	Iowa Women’s Health Study (IWHS)	55–69	20	471/23356(Type I) 71/23356(Type II)	8	≥4 cups/day vs. never or ≤1 cups/month	0.71(0.51–0.99) (Type I) 0.84(0.33–2.12) (Type II)	age, diabetes, duration of HT use, hypertension, age at menarche, age at menopause, BMI, waist-to-hip ratio, smoking status, pack years of smoking, total energy and alcohol use.
Gavrilyuk,2014	Norway	Norwegian Women and Cancer study(NOWAC)	30–70	18	462/ 97926	8	≥ 8 cups/day vs. ≤ 1 cups/day	0.52 (0.34–0.79)	parity, smoking status, BMI, duration of OC and HRT use
Weiderpass,2014	Sweden	Swedish Women’s Lifestyle and Health	30–49	10.9	144/ 42270	9	>3cups/day vs. <2 cups/day	0.64(0.39–1.06)	age, education, duration of hormonal contraceptive use, parity, duration of breastfeeding, smoking status and number of cigarettes/day, menopausal status, BMI, and diabetes mellitus
Merritt,2015	Europe	European Prospective Investigation into Cancer and Nutrition study (EPIC)	25–70	11	1303/301107	9	750.0 g/day vs.8.6 g/day	0.81(0.68–0.97)	Age, BMI, total energy intake, smoking status, age at menarche, oral contraceptive use, menopausal status, postmenopausal hormone use, parity, the age of recruitment and the study center.
Owenyang,2015	United Kingdom	UK Million Women Study	NA	9.3	4067/560356	8	≥5cups/day vs.1-2cups/day	0.92(0.82–1.03)	age, region, socioeconomic status, height, age at menarche, parity, duration of oral contraceptive use, age and status of menopause at study baseline, duration of HT for menopause, BMI, smoking, alcohol consumption, strenuous exercise, and other non-alcoholic fluid intake.

NOS: Newcastle-Ottawa Scale, USA: the United States of America, UK: United Kingdom, NA: not available, BMI: Body Mass Index, HT: Hormone therapy.

**Table 2:** Meta-analysis of intake of coffee, caffeine, different types of coffee and risk of EC (highest vs lowest coffee intake).

**Variables**	**Number of**	**Test of association**		**Test of heterogeneity**		**Publication bias**		P* (value)
	**comparisons**	**Pooled RR(95% CI)**	**P value**	**I2 (%)**	**P value**	**Begg’s test**	**Egger’s test**	
Total coffee	14	0.80(0.74–0.86)	0.00	31	0.13	0.66	0.03	
Caffeine intake	4	0.77(0.65–0.92)	0.00	0	0.87	0.75	0.61	
Type of coffee								0.35
Caffeinated coffee	5	0.66(0.52–0.85)	0.00	53	0.07	0.23	0.47	
Decaffeinated coffee	5	0.77(0.63–0.94)	0.01	0	0.76	1.00	0.88	

**Table 3:** Subgroup analysis to investigate differences between studies included in the meta-analysis (highest vs. lowest coffee intake).

**Variables**	**Number of comparisons**	**Test of association**		**Test of heterogeneity**		**Publication bias**		P*(value)
		**Pooled RR(95% CI)**	**P value**	**I**^**2**^ **(%)**	**P value**	**Begg’s P value**	**Egger’s P value**	
Study location								0.14
Europe	8	0.80(0.71–0.91)	0.00	26	0.22	0.90	0.09	
United States	5	0.72(0.63–0.82)	0.00	0	0.62	1.00	0.86	
Asia	1	0.38(0.16–0.90)	0.03	NA	NA	NA	NA	
Body Mass Index								
<25 kg/m^2^	7	0.99(0.86–1.15)	0.94	0	0.58	0.07	0.03	0.00
≥25 kg/m^2^	4	0.57(0.46–0.71)	0.00	0	0.48	1.00	0.84	
Smoking status								0.68
Never	7	0.66(0.55–0.79)	0.00	0	0.67	0.13	0.00	
Ever	6	0.70(0.56–0.88)	0.00	37	0.16	0.71	0.99	
Hormone therapy								0.04
Never	4	0.60(0.50–0.72)	0.00	0	0.72	1.00	0.44	
Ever	3	0.85(0.65–1.11)	0.24	0	0.92	1.00	0.80	
Menopausal status								0.30
postmenopausal only	4	0.72(0.60–0.88)	0.00	14	0.001	0.75	0.78	
both	10	0.81(0.75–0.88)	0.00	37	0.12	0.72	0.06	
Length of follow-up								0.77
≤15 years	9	0.82(0.75–0.89)	0.00	52	0.03	0.47	0.11	
>15 years	5	0.73(0.63–0.85)	0.00	0	0.98	0.81	0.80	
Number of participants								0.57
<50 000	7	0.77(0.64–0.93)	0.01	0	0.96	0.44	0.12	
>50 000	7	0.72(0.60–0.85)	0.00	72	0.00	0.23	0.00	
Number of cases								0.55
<200	6	0.67(0.49–0.92)	0.01	0	0.74	1.00	0.87	
>200	8	0.75(0.65–0.86)	0.00	64	0.00	0.26	0.02	
Adjust for BMI								0.95
Yes	12	0.74(0.65–0.84)	0.00	52	0.02	0.73	0.04	
NO	2	0.76(0.34–1.68)	0.49	0	0.72	1.00	0.72	
Adjust for hormone therapy								0.74
Yes	10	0.73(0.63–0.84)	0.00	60	0.00	0.37	0.02	
NO	4	0.76(0.61–0.96)	0.02	0	0.96	0.75	0.99	

P* was utilized to assess the subgroup differences.

**Table 4:** Assessment of quality using the GRADE system.

Outcome	Quality assessment								Quality	Importance
	No. of studies	Design	Risk of bias	Inconsistency	Indirectness	Imprecision	Publication bias	Other considerations		
Total coffee intake and EC incidence	11	cohort study	no serious risk of bias	no serious inconsistency	no serious indirectness	no serious imprecision	detected^1^	dose response gradient^2^	low	critical
Caffeine dosage and EC incidence	3	cohort study	no serious risk of bias	no serious inconsistency	no serious indirectness	no serious imprecision	undetected	dose response gradient^3^	moderate	important
Caffeinated coffee intake and EC incidence	4	cohort study	no serious risk of bias	serious inconsistency^4^	no serious indirectness	no serious imprecision	undetected	dose response gradient^5^	low	important
Decaffeinated coffee intake and EC incidence	4	cohort study	no serious risk of bias	no serious inconsistency	no serious indirectness	no serious imprecision	undetected	dose response gradient^6^	moderate	important

^1^We conducted Egger’s test to detect the publication bias; the p value was 0.03.

^2^We conducted dose-response analysis of total coffee consumption and EC, and the result showed that increased intake of1 cup of coffee per day was associated with 5% lower risk of EC.

^3^We conducted dose-response analysis of caffeine dosage and EC, and the result showed that increased intake of 100 mg caffeine per day was associated with a 4% lower risk of EC.

^4^Moderate heterogeneity was detected (P = 0.08, I2 = 52%).

^5^We conducted dose-response analysis of caffeinated coffee consumption and EC, and the result showed that increased intake of 1 cup of coffee per day was associated with a 7% lower risk of EC.

^6^We conducted dose-response analysis of decaffeinated coffee consumption and EC, and the result showed that increased intake of 1 cup of coffee per day was associated with a 4% lower risk of EC.
