# Supplementary material for: Coffee consumption and risk of endometrial cancer: a dose-response meta-analysis of prospective cohort studies
Source: Sci Rep. 2015 Aug 25;5:13410. doi: 10.1038/srep13410 (PMC4548216; doi:10.1038/srep13410)
Supplement: Supplementary Information [file srep13410-s1.pdf]

Coffee consumption and risk of endometrial cancer: a dose-response meta-analysis of prospective cohort studies

Quan Zhou[1], Meilin Luo[2], Hui Li[3], Min Li[4], Jian-Guo Zhou[5]

Appendix 1

Quality assessment according to the Newcastle-Ottawa scale.

Study	Country	Selection	Comparability	Outcome	Score
Jacobsen,1986	Norway	4	0	3	7
Stensvold,1994	Norway	2	0	3	6
Shimazu,2008	Japan	4	2	3	9
Friberg,2009	Sweden	3	0	3	6
Nilsson,2010	Sweden	4	1	2	7
Giri,2011	USA	4	2	2	8
Je,2011	USA	2	2	3	7
Gunter,2012	USA	2	2	3	7
Uccella,2013	USA	4	2	2	8
Gavrilyuk,2014	Norway	4	1	3	8
Weiderpass,2014	Sweden	4	2	3	9
Merritt,2015	Europe	4	2	3	9
Owenyang,2015	United Kingdom	4	2	2	8

Appendix 2

Power calculations: The methodology use is described by Hedges 2001[1] and this macro was obtained from the supplementary material in Cafri 2009[2]. The SAS code for power calculations is below:

Power calculation for meta-analysis of total coffee intake and risk of endometrial cancer(highest versus lowest category)

```
data coffee1;
input es v;
cards;
-0.653926467  1.205550833
-0.223143551  0.192672522
-0.967584026  0.196635863
-0.287682072  0.017211028
-0.127833372  0.127112971
-0.15082289   0.025628571
-0.287682072  0.018394813
```

```
-0.446287103  0.013189878
-0.342490309  0.028631249
-0.174353387  0.225159622
-0.446287103  0.065061102
-0.653926467  0.046256523
-0.210721031  0.00821073
-0.083381609  0.003383255
;
run;
%metapower (test='M', model='fixed', raw_data='yes', alpha=.05,
tau2=99,heterogeneity=99, n1=99, n2=99, k=99, eff_type='or',
T= -0.356674944, Dataset=coffee1, B=NA, v=v, x=NA, es=es, p=NA,
weight=NA);
run;
```

Power calculation for meta-analysis of caffeine dosage and risk of endometrial cancer(highest versus lowest category)

```
data coffee2;
input es v;
cards;
-0.328504067  0.019439167
-0.210721031  0.019194016
-0.020202707  0.176308224
-0.274436846  0.045146398
;
run;
%metapower (test='M', model='fixed', raw_data='yes', alpha=.05,
tau2=99,heterogeneity=99, n1=99, n2=99, k=99, eff_type='or',
T= -0.261364764, Dataset=coffee2, B=NA, v=v, x=NA, es=es, p=NA,
weight=NA);
run;
```

Power calculation for meta-analysis of caffeinated coffee intake and risk of endometrial cancer(highest versus lowest category)

```
data coffee3;
input es v;
cards;
-0.116533816  0.031983705
-0.356674944  0.02518142
-0.776528789  0.024755988
-0.430782916  0.026529825
-0.162518929  0.17755308
```

```
;
run;
%metapower (test='M', model='random', raw_data='yes', alpha=.05,
tau2=99,heterogeneity=99, n1=99, n2=99, k=99, eff_type='or',
T= -0.415515444, Dataset=coffee3, B=NA, v=v, x=NA, es=es, p=NA,
weight=NA);
run;
```

Power calculation for meta-analysis of decaffeinated coffee intake and risk of endometrial cancer(highest versus lowest category)

```
data coffee4;
input es v;
cards;
-0.673344553  0.130456071
-0.248461359  0.026578977
-0.210721031  0.041494074
-0.274436846  0.045146398
0.076961041   0.240204395
;
run;
%metapower (test='M', model='fixed', raw_data='yes', alpha=.05,
tau2=99,heterogeneity=99, n1=99, n2=99, k=99, eff_type='or',
T= -0.261364764, Dataset=coffee4, B=NA, v=v, x=NA, es=es, p=NA,
weight=NA);
run;
```

Power calculation for meta-analysis of boiled coffee intake and risk of endometrial cancer(highest versus lowest category)

```
data coffee5;
input es v;
cards;
-0.274436846  0.264202037
-0.798507696  0.160530659;
run;
%metapower (test='M', model='fixed', raw_data='yes', alpha=.05,
tau2=99,heterogeneity=99, n1=99, n2=99, k=99, eff_type='or',
T= -0.597837001, Dataset=coffee5, B=NA, v=v, x=NA, es=es, p=NA,
weight=NA);
run;
```

Power calculation for meta-analysis of filtered coffee intake and risk of endometrial cancer(highest versus lowest category)

```
data coffee6;
input es v;
cards;
0.104360015   0.099021256
-0.597837001  0.274887469
;
run;
%metapower (test='M', model='random', raw_data='yes', alpha=.05,
tau2=99,heterogeneity=99, n1=99, n2=99, k=99, eff_type='or',
T= -0.287682072, Dataset=coffee6, B=NA, v=v, x=NA, es=es, p=NA,
weight=NA);
run;
```

References

1 Hedges, L. V. & Pigott, T. D. The power of statistical tests in meta-analysis. *Psychological methods* **6**, 203-217 (2001).

2 Cafri, G., Kromrey, J. D. & Brannick, M. T. A SAS macro for statistical power calculations in meta-analysis. *Behavior research methods* **41**, 35-46, doi:10.3758/BRM.41.1.35 (2009).
